# Derivatives of 4-methyl-1,2,3-benzoxathiazine 2,2-dioxide as selective inhibitors of human carbonic anhydrases IX and XII over the cytosolic isoforms I and II

**DOI:** 10.1080/14756366.2023.2170370

**Published:** 2023-01-31

**Authors:** Jekaterīna Ivanova, Morteza Abdoli, Alessio Nocentini, Raivis Žalubovskis, Claudiu T. Supuran

**Affiliations:** a Latvian Institute of Organic Synthesis, Riga, Latvia; b Institute of Technology of Organic Chemistry, Faculty of Materials Science and Applied Chemistry, Riga Technical University, Riga, Latvia; c Neurofarba Department, Università degli Studi di Firenze, Florence, Italy

**Keywords:** Carbonic anhydrase, 123-benzoxathiazine 22-dioxide, 4-methyl-123-benzoxathiazine 22-dioxide, isoform-selective inhibitor

## Abstract

A series of 4-methyl-1,2,3-benzoxathiazine-2,2-dioxides with various substituents in 5, 6 or 7 positions was obtained from corresponding 2’-hydroxyacetophenones in their reaction with sulphamoyl chloride. 6- and 7-aryl substituted 4-methyl-1,2,3-benzoxathiazine-2,2-dioxides were obtained from aryl substituted 2’-hydroxyacetophenonesprepared from 4- or 5-bromo-2’-hydroxyacetophenones via two-step protocol. 4-Methyl-1,2,3-benzoxathiazine-2,2-dioxides were investigated as inhibitors of four human (h) carbonic anhydrase (hCA, EC 4.2.1.1) isoforms, off-target cytosolic hCA I and II, and target transmembrane, tumour-associated hCA IX and XII. Twenty derivatives of 4-methyl-1,2,3-benzoxathiazine 2,2-dioxide were obtained. With one exception (compound**2a**), they mostly act as nanomolar inhibitors of target hCA IX and XII. Basically, all screened compounds express none or low inhibitory properties towards off-target hCA I. hCA II is inhibited in micromolar range. Overwhelming majority of 4-methyl-1,2,3-benzoxathiazine 2,2-dioxides express excellent selectivity towards CA IX/XII over hCA I as well as very good selectivity towards CA IX/XII over hCA II.

## Introduction

Carbonic anhydrases (CAs, EC 4.2.1.1) are ubiquitous metalloenzymes found in all living organisms. They catalyse the reversible hydration of carbon dioxide to bicarbonate and proton. This physiological reaction is essential for variety of normal and pathological processes such as CO_2_ and pH homeostasis, respiration, gluconeogenesis, calcification, bone resorption, fluid secretion and tumorigenesis^1–7^.

Sixteen different α-CA isoforms have been identified in mammals so far^1^^,^^3^. hCA I and II are widely distributed isoforms which may serve as targets for some diseases and off-targets for others^7^. hCA IX and XII are two transmembrane, multi-domain, hypoxia-induced tumour-associated proteins discovered by Pastorek et al.^8^ in 1994 and Türeci et al.^9^ in 1998 that have received attention as diagnostic markers and potential drug targets for cancer^10^^,^^11^.

Tumour growth, angiogenesis, proliferation and metastasis are ascribed to the overexpressed levels of hCA IX and XII. This can be used as a strategy for targeting of these enzymes as a new approach in cancer treatment. Selective inhibition of the tumour-associated hCA IX and XII over the other isoforms, especially the most prevalent cytosolic hCA I and II is important and will result in cancer treatment with fewer side effects^7^.

hCA IX is not significantly expressed in the majority of normal tissues and is present only in the stomach and gallbladder epithelia, but is overexpressed in variety of solid tumors^10^^,^^11^. On the other hand, hCA XII is abundant in many healthy tissues, like kidney, prostate, pancreas, intestine and lymphocytes, but overexpressed in a certain number of malignant tumours, and associated with less-aggressive, well-differentiated tumour phenotypes as compared to the hCA IX expressing tumours thus CA IX is the preferred isoform for pharmacological intervention^7^^,^^10–12^. hCA IX has been considered as a valuable marker for cancer, and the development of hCA IX inhibitors with selectivity over ubiquitous isoforms hCA I/II is a potential strategy for designing anticancer agents^3^^,^^12^.

**Figure 1.**
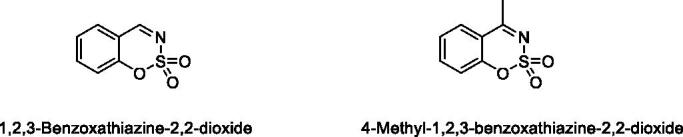
Structures of 1,2,3-benzoxathiazine-2,2-dioxide and its methyl derivative.

Classical CA inhibitors (CAIs) usually have a sulphonamide moiety as a zinc-binding group (ZBG), such as clinically used CAIs acetazolamide and methazolamide. On the other hand, the non-classical CAIs do not rely on ZBG^13^^,^^14^. Non-classical CAIs such as coumarins, carboxylic acids, phenols, polyamines inhibit the catalytic activity of CA by different mechanisms rather than coordinating to the zinc^12^. Coumarin derivatives were first reported as a non-classical type of CAIs in 2009 by Supuran and coworkers^15^. Since then a number of structurally similar compounds (coumarin derivatives and their bioisosteres) were reported as CAIs, such as sulphocoumarins^16–22^, 3*H*-1,2-benzoxathiepine 2,2-dioxides^23–26^, benzoxepinones^27^, isocoumarins^28^. In our previous work we described the synthesis and CA inhibitory activity of new class of CAIs – nitrogen-containing analogues of 1,2-benzoxathiine 2,2-dioxides, namely 1,2,3-benzoxathiazine-2,2-dioxides^29^. In this work we continue the research and extend the range of 1,2,3-benzoxathiazine-2,2-dioxide derivatives focussing on 4-methyl-1,2,3-benzoxathiazine-2,2-dioxides to provide more data about their CA inhibition properties (Figure 1).

## Materials and methods

### Chemistry

Reagents, starting materials and solvents were obtained from commercial sources and used as received. Thin-layer chromatography was performed on silica gel, spots were visualised with UV light (254 and 365 nm). Melting points were determined on an OptiMelt automated melting point system. IR spectra were recorded on Shimadzu FTIR IR Prestige-21 spectrometer. NMR spectra were recorded on Bruker Avance Neo (400 MHz) spectrometer with chemical shifts values (δ) in ppm relative to TMS using the residual CDCl_3_ signal (^1^H 7.26; ^13^C 77.16) as an internal standard. High-resolution mass spectra (HRMS) were recorded on a mass spectrometer with a Q-TOF micro mass analyser using the ESI technique. Elemental analyses were performed on Carlo Erba CHNS-O EA-1108 apparatus. GC-MS analyses were performed on *Agilent Technologies* 7890 A gas chromatograph, column – *HP-5HS* (df = 0,25 μm, ID = 0,25 mm, length − 30 m) with *Agilent Technologies* 5975 C masselective detector.

#### Sulphamoyl chloride^30^


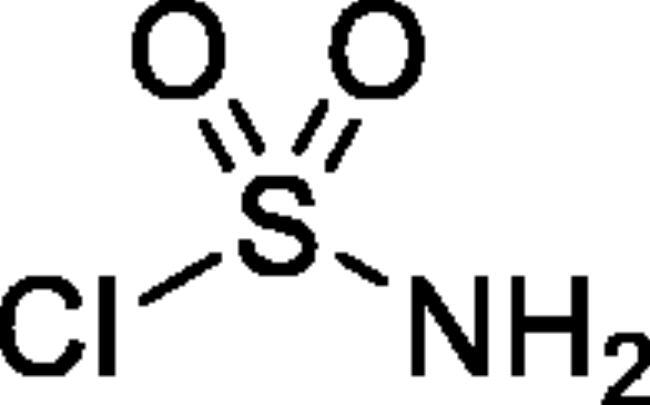


Chlorosulphonylisocyanate (3.80 ml, 43.3 mmol) was cooled to +5 °C. Formic acid (1.67 ml, 43.3 mmol) was added dropwise in temperature range between +5 and +13 °C. Reaction mixture was stirred at 0 °C for additional 45 min, then allowed to warm to room temperature. Toluene (30 ml) was added to the reaction mixture, precipitate was filtered, and filtrate was evaporated. Product was obtained as yellow oily solid (6.58 g, 98%). Mp 36–37 °C.

#### General procedure for the preparation of 1,2,3-benzoxathiazine 2,2-dioxides 2a-j

The derivative of acetophenone (1 equiv) was dissolved in dry DMA (6 ml). Reaction mixture was cooled to 0 °C. Sulphamoyl chloride (2.5 equiv) was slowly added to the reaction mixture. The stirring was continued at room temperature under argon atmosphere for 24–72 h. Reaction mixture was then poured into ice-water (25 ml), extracted with DCM (3 × 25 ml), washed with satd. NaHCO_3_ (3 × 25 ml) and satd. NaCl (3 × 25 ml). Organic phase was dried over Na_2_SO_4_, filtered, evaporated. Product was purified by column chromatography on silica gel with PE/EtOAc (2:1) followed by recrystallization from EtOH.

#### 5-Methoxy-4-methyl-1,2,3-benzoxathiazine 2,2-dioxide (2a)^31^


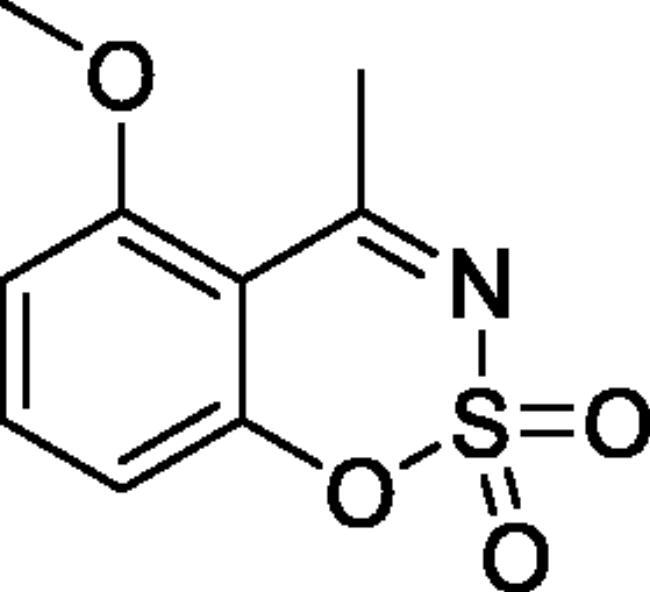


Obtained from 1–(2-hydroxy-6-methoxyphenyl)ethanone (0.20 g, 1.20 mmol) and sulphamoyl chloride (0.35 g, 3.00 mmol). Reaction mixture was stirred for 72 h. Purified by column chromatography with PE/EtOAc (1:1). Obtained as light yellow solid (0.12 g, 44%). Mp 91–92 °C.

IR (KBr) cm^−1^ = 1374 (S = O), 1194 (S = O). ^1^H NMR (400 MHz, CDCl_3_) *δ* = 7.60 (1H, t, *J*** **=** **8.4 Hz), 6.85–6.89 (2H, *m*), 3.99 (3H, *s*), 2.77 (3H, *s*) ppm ^13^C NMR (100 MHz, CDCl_3_)*δ* = 178.7, 160.0, 154.6, 137.2, 111.1, 108.8, 56.6, 29.2 ppm Analysis calculated for C_9_H_9_NO_4_S (227.23): C, 47.57; H, 3.99; N 6.16. Found: C, 48.17; H, 4.14; N, 6.02. GC-MS (m/z, %): 120 (60), 133 (37), 134 (24), 162 (26), 227 (100).

#### 4,6-Dimethyl-1,2,3-benzoxathiazine 2,2-dioxide (2 b)^32^


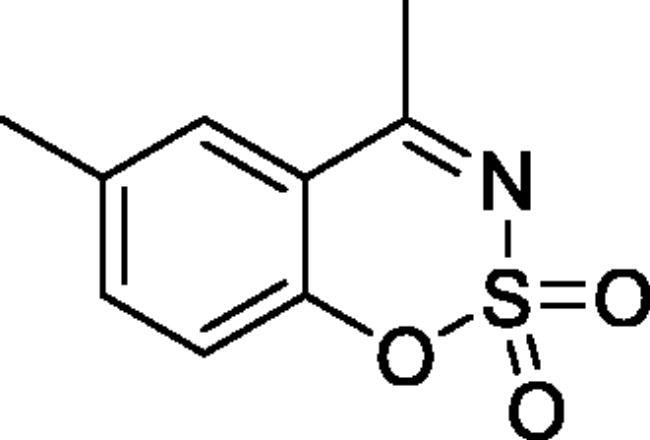


Obtained from 1–(2-hydroxy-5-methylphenyl)ethanone (0.20 g, 1.33 mmol) and sulphamoyl chloride (0.38 g, 3.33 mmol). Reaction mixture was stirred for 72 h. Obtained as white solid (0.18 g, 65%). Mp 125–126 °C. IR (KBr) cm^−1^= 1373 (S = O), 1187 (S = O). ^1^H NMR (400 MHz, CDCl_3_) *δ* = 7.55–7.58 (1H, m), 7.49–7.53 (1H, *m*), 7.18 (1H, d, *J*** **=** **8.4 Hz), 2.70 (3H, *s*), 2.44 (3H, *s*) ppm ^13^C NMR (100 MHz, CDCl_3_)*δ* = 177.6, 151.7, 138.1, 136.2, 128.6, 119.0, 116.4, 23.9, 21.1 ppm. Analysis calculated for C_9_H_9_NO_3_S (211.24): C, 51.17; H, 4.29; N 6.63. Found: C, 51.46; H, 4.56; N, 6.47. GC-MS (m/z, %): 77 (27), 78 (57), 106 (20), 118 (31), 146 (54), 211 (100).

#### 6-Fluoro-4-methyl-1,2,3-benzoxathiazine 2,2-dioxide (2c)^32^


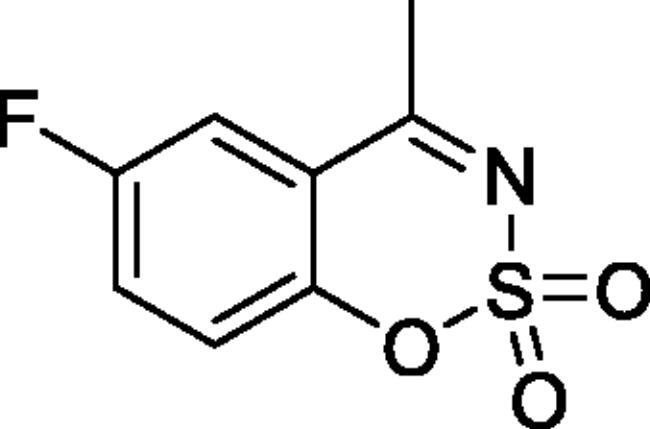


Obtained from 1–(5-fluoro-2-hydroxyphenyl)ethanone (0.20 g, 1.30 mmol) and sulphamoylchloride (0.38 g, 3.25 mmol). Reaction mixture was stirred for 72 h. Obtained as white solid (0.15 g, 55%). Mp 147–148 °C. IR (KBr) cm^−1^ = 1379 (S = O), 1199 (S = O), 1178 (S = O). ^1^H NMR (400 MHz, CDCl_3_) *δ* = 7.40–7.50 (2H, *m*), 7.30 (1H, dd, *J*** **=** **9.0, 4.3 Hz), 2.71 (3H, *s*) ppm. ^13^C NMR (100 MHz, CDCl_3_) *δ* = 176.4 (d, *J*** **=** **2.3 Hz), 159.1 (d, *J*** **=** **248.4 Hz), 149.6 (d, *J*** **=** **2.5 Hz), 124.4 (d, *J*** **=** **24.0 Hz), 121.1 (d, *J*** **=** **8.1 Hz), 117.2 (d, *J*** **=** **7.7 Hz), 114.6 (d, *J*** **=** **25.1 Hz), 23.9 ppm. Analysis calculated for C_8_H_6_FNO_3_S (215.20): C, 44.65; H, 2.81; N 6.51. Found: C, 44.73; H, 2.82; N, 6.40. GC-MS (m/z, %): 81 (24), 82 (99), 96 (30), 108 (28), 122 (33), 151 (46), 215 (100).

#### 6-Chloro-4-methyl-1,2,3-benzoxathiazine 2,2-dioxide (2d)^32^


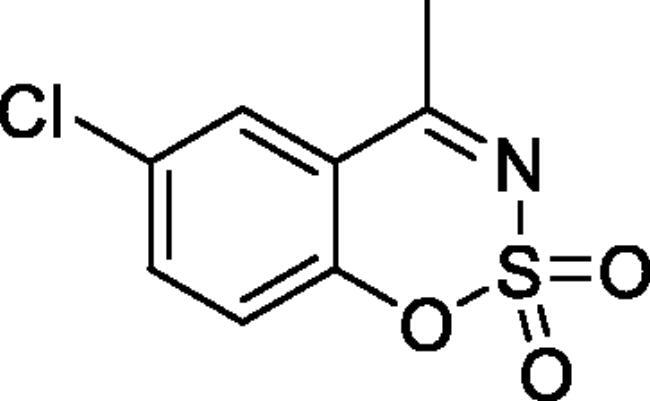


Obtained from 1–(5-chloro-2-hydroxyphenyl)ethanone (0.20 g, 1.17 mmol) and sulphamoyl chloride (0.34 g, 2.93 mmol). Reaction mixture was stirred for 72 h. Obtained as white solid (0.19 g, 69%). Mp 151–152 °C. IR (KBr) cm^−1^= 1375 (S = O), 1186 (S = O). ^1^H NMR (400 MHz, CDCl_3_) *δ* = 7.76 (1H, d, *J*** **=** **2.4 Hz), 7.67 (1H, dd, *J*** **=** **8.8, 2.4 Hz), 7.26 (1H, d, *J*** **=** **8.8 Hz), 2.72 (3H, *s*) ppm. ^13^C NMR (100 MHz, CDCl_3_) *δ* = 176.3, 152.0, 137.0, 131.5, 128.1, 120.8, 117.4, 23.9 ppm. Analysis calculated for C_8_H_6_ClNO_3_S (231.65): C, 41.48; H, 2.61; N 6.05. Found: C, 41.63; H, 2.60; N, 5.92. GC-MS (m/z, %): 63 (54), 98 (33), 104 (44), 124 (23), 167 (64), 169 (21), 231 (100).

#### 6-Bromo-4-methyl-1,2,3-benzoxathiazine 2,2-dioxide (2e)^32^


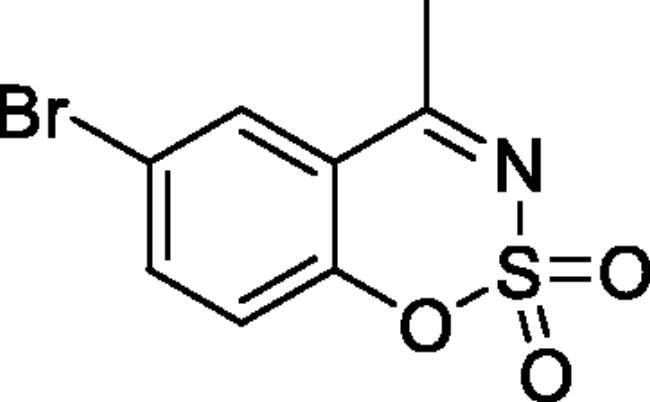


Obtained from 1–(5-bromo-2-hydroxyphenyl)ethanone (0.20 g, 0.93 mmol) and sulphamoyl chloride (0.27 g, 2.33 mmol). Reaction mixture was stirred for 72 h. Purified by column chromatography with PE/EtOAc (4:1). Obtained as white solid (0.06 g, 23%). Mp 153–154 °C. IR (KBr) cm^−1^ = 1385 (S = O), 1193 (S = O). ^1^H NMR (400 MHz, CDCl_3_) *δ* = 7.91 (1H, d, *J*** **=** **2.3 Hz), 7.81 (1H, dd, *J*** **=** **8.8, 2.3 Hz), 7.20 (1H, d, *J*** **=** **8.8 Hz), 2.72 (3H, *s*) ppm. ^13^C NMR (100 MHz, CDCl_3_)*δ* = 176.2, 152.5, 139.9, 131.1, 121.0, 118.6, 117.8, 23.9 ppm. Analysis calculated for C_8_H_6_BrNO_3_S (276.10): C, 34.80; H, 2.19; N 5.07. Found: C, 37.04; H, 2.77; N, 4.77. GC-MS (*m/z*, %): 62 (24), 63 (74), 104 (43), 170 (21), 211 (39), 277 (100).

#### 4,7-Dimethyl-1,2,3-benzoxathiazine 2,2-dioxide (2f)^32^


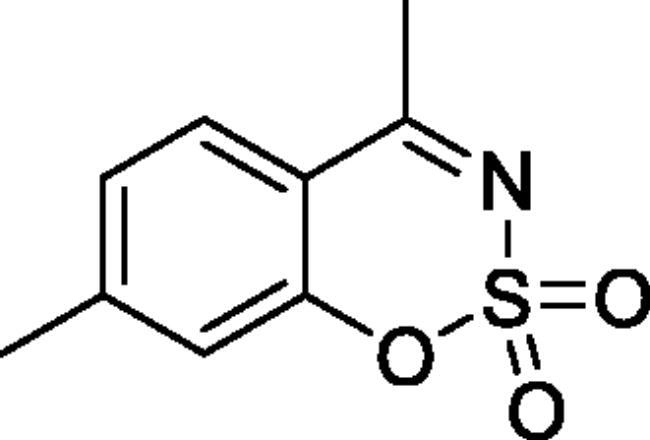


Obtained from 1–(2-hydroxy-4-methylphenyl)ethanone (0.19 ml, 0.20 g, 1.33 mmol) and sulphamoyl chloride (0.38 g, 3.33 mmol). Reaction mixture was stirred for 48 h. Obtained as white solid (0.18 g, 63%). Mp 134–135 °C. IR (KBr) cm^−1^ = 1378 (S = O), 1368 (S = O), 1189 (S = O). ^1^H NMR (400 MHz, CDCl_3_) *δ* = 7.66 (1H, d, *J*** **=** **8.1 Hz), 7.16–7.20 (1H, *m*), 7.08–7.11 (1H, *m*), 2.69 (3H, *s*), 2.48 (3H, *s*) ppm. ^13^C NMR (100 MHz, CDCl_3_) *δ* = 177.3, 153.7, 149.7, 128.4, 127.0, 119.3, 114.3, 23.7, 22.2 ppm. Analysis calculated for C_9_H_9_NO_3_S (211.24): C, 51.17; H, 4.29; N 6.63. Found: C, 51.21; H, 4.36; N, 6.57. GC-MS (*m/z*, %): 77 (20), 78 (39), 118 (43), 146 (35), 211 (100).

#### 7-Methoxy-4-methyl-1,2,3-benzoxathiazine 2,2-dioxide (2 g)^33^


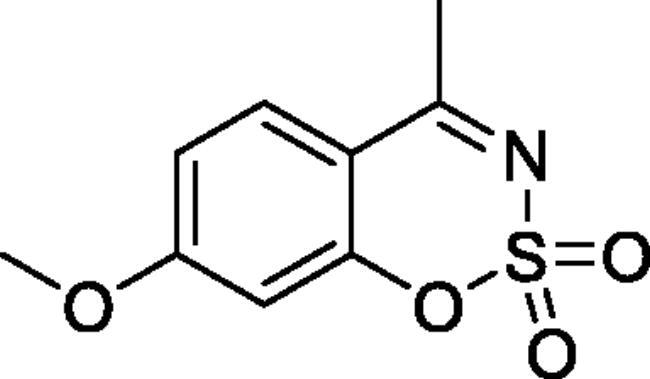


Obtained from 1–(2-hydroxy-4-methoxyphenyl)ethanone (0.20 g, 1.20 mmol) and sulphamoyl chloride (0.35 g, 3.00 mmol). Reaction mixture was stirred for 72 h. Obtained as white solid (0.08 g, 30%). Mp 163–164 °C. IR (KBr) cm^−1^ = 1384 (S = O), 1352 (S = O), 1186 (S = O). ^1^H NMR (400 MHz, CDCl_3_) *δ* = 7.69 (1H, d, *J*** **=** **8.9 Hz), 6.87 (1H, d, *J*** **=** **8.9, 2.5 Hz), 6.73 (1H, d, *J*** **=** **2.5 Hz), 3.92 (3H, *s*), 2.66 (3H, *s*) ppm. ^13^C NMR (100 MHz, CDCl_3_) *δ* = 176.7, 166.7, 156.1, 130.2, 113.5, 110.0, 103.0, 56.4, 23.7 ppm. Analysis calculated for C_9_H_9_NO_4_S (227.23): C, 47.57; H, 3.99; N 6.16. Found: C, 47.90; H, 4.08; N, 6.04. GC-MS (*m/z*, %): 106 (21), 120 (32), 148 (61), 227 (100).

#### 7-Fluoro-4-methyl-1,2,3-benzoxathiazine 2,2-dioxide (2 h)^33^


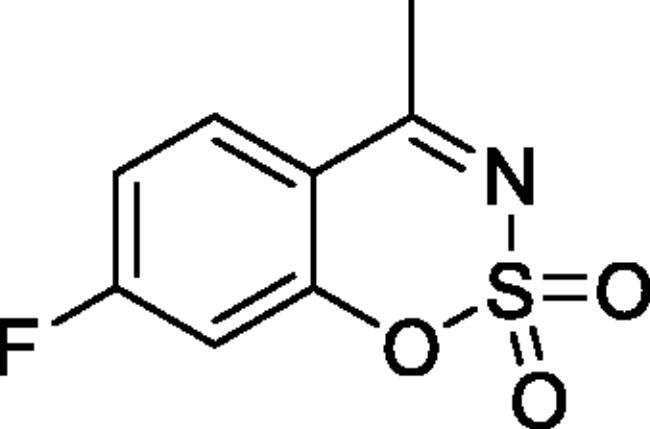


Obtained from 1–(4-fluoro-2-hydroxyphenyl)ethanone (0.20 g, 1.30 mmol) and sulphamoyl chloride (0.38 g, 3.25 mmol). Reaction mixture was stirred for 72 h. Obtained as white solid (0.07 g, 24%). Mp 107–108 °C. IR (KBr) cm^−1^ = 1380 (S = O), 1374 (S = O), 1123 (S = O). ^1^H NMR (400 MHz, CDCl_3_) *δ* = 7.83 (1H, dd, *J*** **=** **8.9, 5.8 Hz), 7.11 (1H, ddd, *J*** **=** **8.9, 7.8, 2.5 Hz), 7.02 (1H, dd, *J*** **=** **8.4, 2.5 Hz), 2.71 (3H, *s*) ppm. ^13^C NMR (100 MHz, CDCl_3_) *δ* = 176.5, 167.2 (d, *J*** **=** **262.6 Hz), 155.4 (d, *J*** **=** **13.4 Hz), 131.0 (d, *J*** **=** **11.1 Hz), 114.1 (d, *J*** **=** **22.4 Hz), 113.4 (d, *J*** **=** **3.4 Hz), 107.1 (d, *J*** **=** **25.9 Hz), 24.0 ppm. Analysis calculated for C_8_H_6_FNO_3_S (215.20): C, 44.65; H, 2.81; N 6.51. Found: C, 44.83; H, 2.89; N, 6.37. GC-MS (*m/z*, %): 82 (26), 96 (29), 108 (24), 123 (36), 151 (32), 215 (100).

#### 7-Chloro-4-methyl-1,2,3-benzoxathiazine 2,2-dioxide (2i)^34^


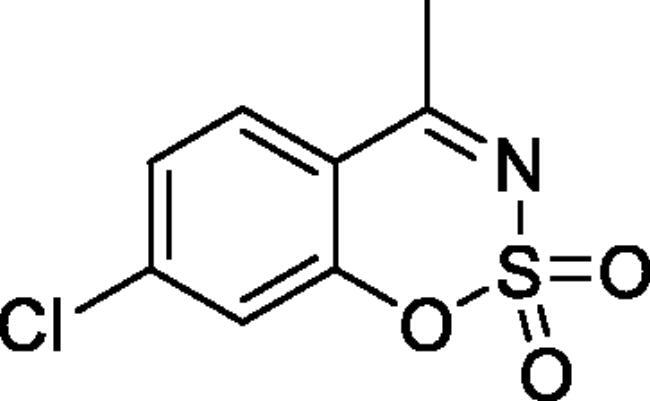


Obtained from 1–(4-chloro-2-hydroxyphenyl)ethanone (0.15 ml, 0.20 g, 1.17 mmol) and sulphamoyl chloride (0.34 g, 2.93 mmol). Reaction mixture was stirred for 72 h. Obtained as white solid (0.13 g, 48%). Mp 160–161 °C. IR (KBr) cm^−1^ = 1383 (S = O), 1375 (S = O), 1193 (S = O). ^1^H NMR (400 MHz, CDCl_3_) *δ* = 7.73 (1H, d, *J*** **=** **8.5 Hz), 7.37 (1H, dd, *J*** **=** **8.5, 2.0 Hz), 7.32 (1H, d, *J*** **=** **2.0 Hz), 2.71 (3H, *s*) ppm. ^13^C NMR (100 MHz, CDCl_3_) *δ* = 176.6, 154.0, 143.3, 129.5, 126.6, 119.6, 115.0, 23.9 ppm. Analysis calculated for C_8_H_6_ClNO_3_S (231.65): C, 41.48; H, 2.61; N 6.05. Found: C, 42.29; H, 2.77; N, 5.88. GC-MS (*m/z*, %): 63 (50), 104 (53), 124 (24), 139 (39), 167 (42), 231 (100), 233 (38).

#### 7-Bromo-4-methyl-1,2,3-benzoxathiazine 2,2-dioxide (2j)^32^


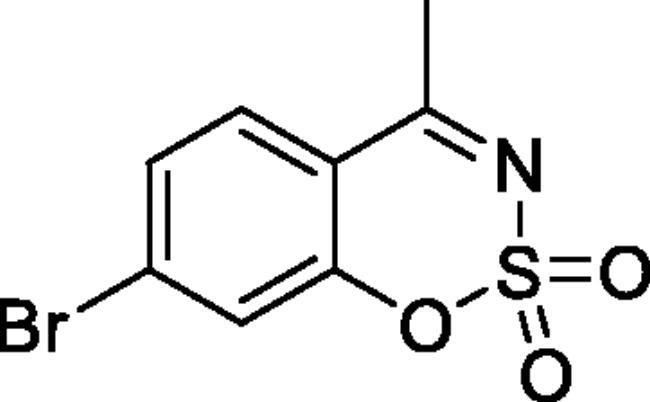


Obtained from 1–(4-bromo-2-hydroxyphenyl)ethanone (0.20 g, 0.93 mmol) and sulphamoyl chloride (0.27 g, 2.33 mmol). Reaction mixture was stirred for 72 h. Obtained as white solid (0.05 g, 21%). Mp 193–194 °C. IR (KBr) cm^−1^ = 1379 (S = O), 1373 (S = O), 1189 (S = O). ^1^H NMR (400 MHz, CDCl_3_) *δ* = 7.65 (1H, d, *J*** **=** **8.5 Hz), 7.53 (1H, dd, *J*** **=** **8.5, 1.8 Hz), 7.49 (1H, d, *J*** **=** **1.8 Hz), 2.70 (3H, *s*) ppm. ^13^C NMR (100 MHz, CDCl_3_) *δ* = 176.7, 153.8, 131.7, 129.5, 129.4, 122.6, 115.4, 23.9 ppm. Analysis calculated for C_8_H_6_BrNO_3_S (276.10): C, 34.80; H, 2.19; N 5.07. Found: C, 35.90; H, 2.41; N, 4.82. GC-MS (m/z, %): 63 (69), 104 (67), 183 (26), 211 (25), 277 (100).

#### General procedure for the preparation of 2’-hydroxyacetophenones 4a-j

The derivative of acetophenone (1 equiv), the derivative of benzeneboronic acid (1.3 equiv), K_2_CO_3_ (2.5 equiv) and Pd(PPh_3_)_4_ (0.05 equiv) were suspended in toluene/water (5:1, 20 ml) mixture in a pressure tube. Reaction mixture was heated to 90 °C and stirred for 24 h, then cooled to room temperature, filtered through celite. EtOAc (30 ml) was added, reaction mixture was washed with NaHCO_3_ (satd., 3 × 25 ml) and brine (2 × 25 ml). Organic phase was dried over Na_2_SO_4_, filtered, evaporated. The product was purified by column chromatography on silica gel with PE/EtOAc (10:1) followed by recrystallization from EtOH.

#### 4-Hydroxy-1,1'-biphenyl-3-carbaldehyde (4a)^35^


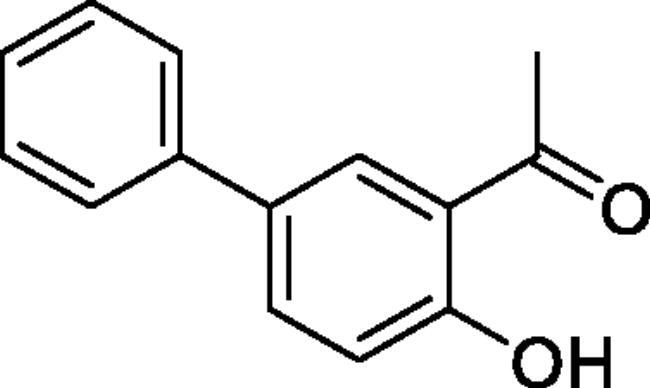


Obtained from 5′-bromo-2′-hydroxyacetophenone (0.50 g, 2.33 mmol), benzeneboronic acid (0.37 g, 3.02 mmol), K_2_CO_3_ (0.80 g, 5.81 mmol) and Pd(PPh_3_)_4_ (0.13 g, 0.12 mmol). Obtained as light brown solid (0.37 g, 74%). Mp 60–61 °C. IR (KBr) cm^−1^ = 3038 (OH), 1643 (C = O). ^1^H NMR (400 MHz, CDCl_3_) *δ* = 12.27 (1H, *s*), 7.92 (1H, d, *J*** **=** **2.3 Hz), 7.72 (1H, dd, *J*** **=** **8.6, 2.3 Hz), 7.52–7.57 (2H, *m*), 7.43–7.49 (2H, *m*), 7.33–7.39 (1H, *m*), 7.07 (1H, d, *J*** **=** **8.6 Hz), 2.70 (3H, *s*) ppm. ^13^C NMR (100 MHz, CDCl_3_) *δ* = 204.7, 161.9, 140.1, 135.5, 132.5, 129.2, 129.1, 127.4, 126.8, 119.9, 119.0, 26.9 ppm. HRMS (ESI) [M + H]^+^: *m*/*z* calcd for (C_14_H_13_O_2_) 213.0916. Found 213.0911. GC-MS (m/z, %): 115 (22), 197 (100), 212 (72).

#### 4'-Fluoro-4-hydroxy-1,1'-biphenyl-3-carbaldehyde (4b)^36^


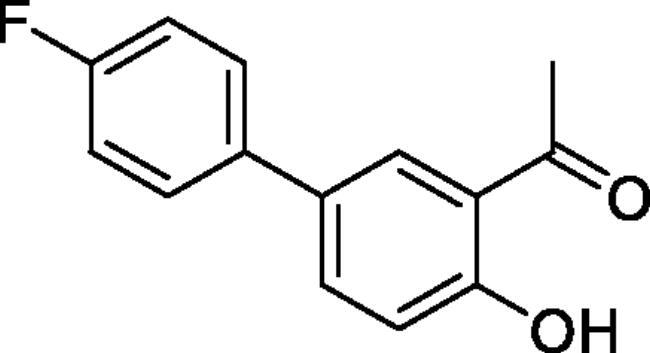


Obtained from 5′-bromo-2′-hydroxyacetophenone (0.50 g, 2.33 mmol), 4-fluorobenzeneboronic acid (0.42 g, 3.02 mmol), K_2_CO_3_ (0.80 g, 5.81 mmol) and Pd(PPh_3_)_4_ (0.13 g, 0.12 mmol). Obtained as light yellow solid (0.45 g, 84%). Mp 129–130 °C. IR (KBr) cm^−1^ = 1640 (C = O). ^1^H NMR (400 MHz, CDCl_3_) *δ* = 12.26 (1H, *s*), 7.85 (1H, d, *J*** **=** **8.6, 2.3 Hz), 7.66 (1H, dd, *J*** **=** **8.6, 2.3 Hz), 7.49 (2H, dd, *J*** **=** **8.9, 5.2 Hz), 7.14 (2H, *t*, *J*** **=** **8.7 Hz), 7.06 (1H, d, *J*** **=** **8.6 Hz), 2.70 (3H, s) ppm. ^13^C NMR (100 MHz, CDCl_3_) *δ* = 204.6, 162.5 (d, *J*** **=** **246.4 Hz), 161.9, 136.3 (d, *J*** **=** **3.3 Hz), 135.4, 131.6, 129.0, 128.4 (d, *J*** **=** **8.1 Hz), 119.9, 119.1, 116.0 (d, *J*** **=** **21.5 Hz), 26.9 ppm. HRMS (ESI) [M + H]^+^: *m*/*z* calcd for (C_14_H_12_O_2_F) 231.0821. Found 231.0816. GC-MS (m/z, %): 159 (21), 215 (100), 230 (75).

#### 4-Hydroxy-4'-methoxy-1,1'-biphenyl-3-carbaldehyde (4c)^36^


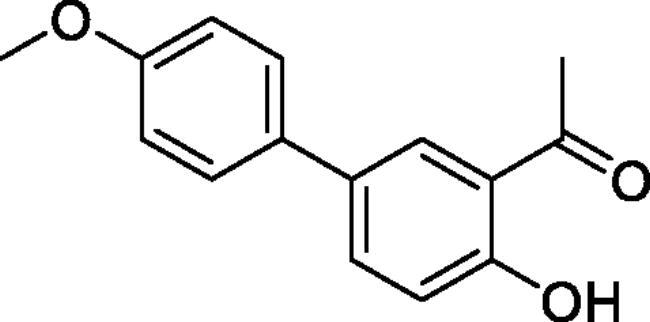


Obtained from 5′-bromo-2′-hydroxyacetophenone (0.50 g, 2.33 mmol), 4-methoxybenzeneboronic acid (0.46 g, 3.02 mmol), K_2_CO_3_ (0.80 g, 5.81 mmol) and Pd(PPh_3_)_4_ (0.13 g, 0.12 mmol). Obtained as white solid (0.50 g, 89%). Mp 112–113 °C. IR (KBr) cm^−1^ = 2958 (OH), 1653 (C = O). ^1^H NMR (400 MHz, CDCl_3_) *δ* = 12.21 (1H, *s*), 7.86 (1H, d, *J*** **=** **2.3 Hz), 7.67 (1H, dd, *J*** **=** **8.6, 2.3 Hz), 7.46 (2H, d, *J*** **=** **8.8 Hz), 7.04 (1H, d, *J*** **=** **8.6 Hz), 6.99 (2H, d, *J*** **=** **8.8 Hz), 3.86 (3H, *s*), 2.69 (3H, *s*) ppm. ^13^C NMR (100 MHz, CDCl_3_) *δ* = 204.7, 161.5, 159.2, 135.2, 132.7, 132.2, 128.7, 127.9, 119.9, 118.9, 114.5, 55.5, 26.9 ppm. HRMS (ESI) [M + H]^+^: *m*/*z* calcd for (C_15_H_15_O_3_) 243.1021. Found 243.1023. GC-MS (*m/z*, %): 227 (63), 242 (100).

#### 3',4'-Dichloro-4-hydroxy-1,1'-biphenyl-3-carbaldehyde (4d)


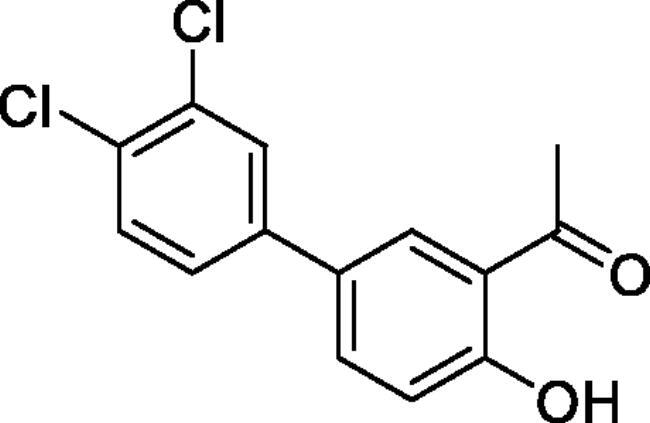


Obtained from 5′-bromo-2′-hydroxyacetophenone (0.50 g, 2.33 mmol), 3,4-dichlorobenzeneboronic acid (0.58 g, 3.02 mmol), K_2_CO_3_ (0.80 g, 5.81 mmol) and Pd(PPh_3_)_4_ (0.13 g, 0.12 mmol). Obtained as white solid (0.39 g, 59%). Mp 145–146 °C. IR (KBr) cm^−1^ = 3060 (OH), 1646 (C = O). ^1^H NMR (400 MHz, CDCl_3_) *δ* = 12.32 (1H, *s*), 7.86 (1H, d, *J*** **=** **2.3 Hz), 7.66 (1H, dd, *J*** **=** **8.7, 2.2 Hz), 7.61 (1H, d, *J*** **=** **2.2 Hz), 7.51 (1H, d, *J*** **=** **8.3 Hz), 7.36 (1H, dd, *J*** **=** **2.2 Hz, *J*** **=** **8.3 Hz), 7.08 (1H, d, *J*** **=** **8. 7 Hz), 2.71 (3H, *s*) ppm. ^13^C NMR (100 MHz, CDCl_3_) *δ* = 204.5, 162.5, 140.1, 135.1, 133.2, 131.5, 131.0, 130.0, 129.1, 128.6, 126.1, 120.0, 119.4, 26.9 ppm. HRMS (ESI) [M - H]^-^: *m*/*z* calcd for (C_14_H_9_O_2_Cl_2_) 278.9980. Found 278.9976. GC-MS (*m/z*, %): 139 (21), 202 (26), 265 (100), 267 (66), 280 (72), 281 (12).

#### Ethyl 3'-formyl-4'-hydroxy-1,1'-biphenyl-4-carboxylate (4e)


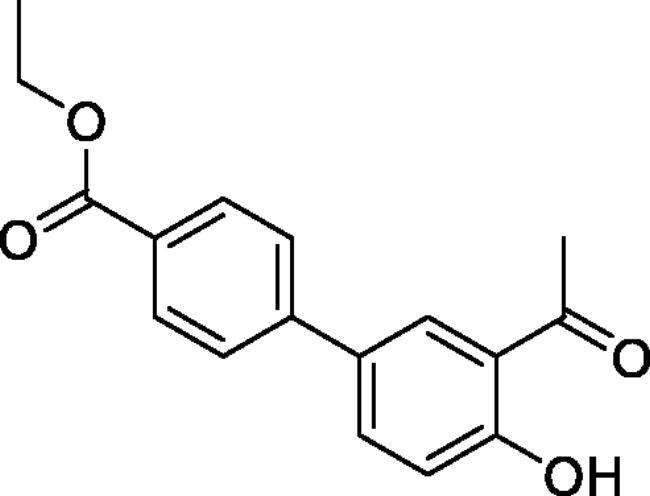


Obtained from 5′-bromo-2′-hydroxyacetophenone (0.50 g, 2.33 mmol), 4-(ethoxycarbonyl)benzeneboronic acid (0.65 g, 3.02 mmol), K_2_CO_3_ (0.80 g, 5.81 mmol) and Pd(PPh_3_)_4_ (0.13 g, 0.12 mmol). Obtained as white solid (0.41 g, 74%). Mp 145–146 °C. IR (KBr) cm^−1^ = 2998 (OH), 1716 (C = O), 1646 (C = O). ^1^H NMR (400 MHz, CDCl_3_) *δ* = 12.37 (1H, s), 8.12 (2H, d, *J*** **=** **8.6 Hz), 7.96 (1H, d, *J*** **=** **2.3 Hz), 7.75 (1H, dd, *J*** **=** **8.6, 2.3 Hz), 7.60 (2H, d, *J*** **=** **8.6 Hz), 7.09 (1H, d, *J*** **=** **8.6 Hz), 4.41 (2H, q, *J*** **=** **7.2 Hz), 2.72 (3H, *s*), 1.42 (3H, *t*, *J*** **=** **7.2 Hz) ppm. ^13^C NMR (100 MHz, CDCl_3_) *δ* = 204.6, 166.5, 162.6, 144.3, 135.4, 131.2, 130.4, 129.4, 129.3, 126.6, 120.0, 119.3, 61.2, 26.9, 14.5 ppm. HRMS (ESI) [M + H]^+^: *m*/*z* calcd for (C_17_H_17_O_4_) 285.1127. Found 285.1127. GC-MS (*m/z*, %): 207 (80), 239 (58), 241 (24), 269 (91), 281 (27), 284 (100).

#### 3-Hydroxy-1,1'-biphenyl-4-carbaldehyde (4f)^37^


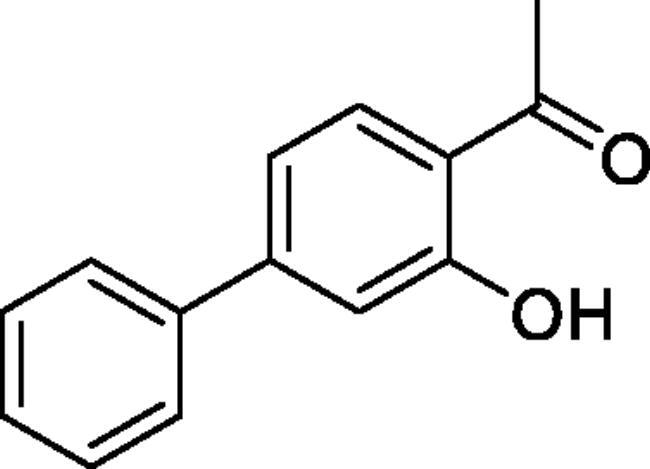


Obtained from 4′-bromo-2′-hydroxyacetophenone (0.50 g, 2.33 mmol), benzeneboronic acid (0.37 g, 3.02 mmol), K_2_CO_3_ (0.80 g, 5.81 mmol) and Pd(PPh_3_)_4_ (0.13 g, 0.12 mmol). Obtained as white solid (0.45 g, 74%). Mp 92–93 °C. IR (KBr) cm^−1^ = 3039 (OH), 1623 (C = O). ^1^H NMR (400 MHz, CDCl_3_) *δ* = 12.36 (1H, *s*), 7.80 (1H, d, *J*** **=** **8.3 Hz), 7.60–7.65 (2H, *m*), 7.38–7.50 (3H, *m*), 7.22 (1H, d, *J*** **=** **1.8 Hz), 7.15 (1H, dd, *J*** **=** **8.3, 1.8 Hz), 2.66 (3H, *s*) ppm. ^13^C NMR (100 MHz, CDCl_3_) *δ* = 204.2, 162.8, 149.3, 139.5, 131.3, 129.1, 128.8, 127.4, 118.7, 118.0, 116.6, 26.8 ppm. HRMS (ESI) [M + H]^+^: *m*/*z* calcd for (C_14_H_13_O_2_) 213.0916. Found 213.0920. GC-MS (*m/z*, %): 197 (100), 212 (45).

#### 4'-Fluoro-3-hydroxy-1,1'-biphenyl-4-carbaldehyde (4g)^38^


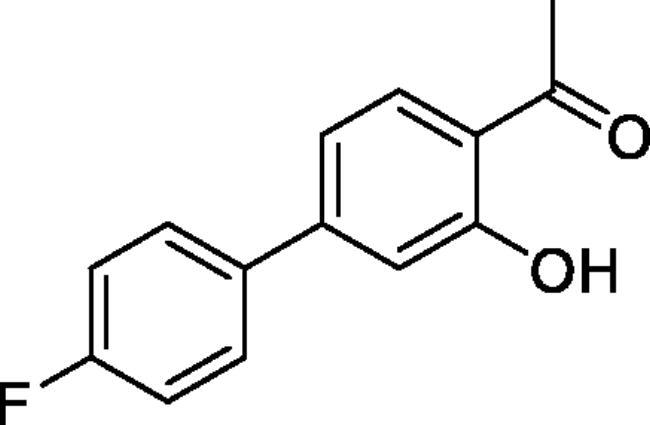


Obtained from 4′-bromo-2′-hydroxyacetophenone (0.50 g, 2.33 mmol), 4-fluorobenzeneboronic acid (0.42 g, 3.02 mmol), K_2_CO_3_ (0.80 g, 5.81 mmol) and Pd(PPh_3_)_4_ (0.13 g, 0.12 mmol). Obtained as light yellow solid (0.31 g, 58%). Mp 94–95 °C. IR (KBr) cm^−1^ = 3071 (OH), 1648 (C = O). ^1^H NMR (400 MHz, CDCl_3_) *δ* = 12.35 (1H, *s*), 7.79 (1H, d, *J*** **=** **8.3 Hz), 7.59 (2H, dd, *J*** **=** **8.9, 5.3 Hz), 7.12–7.18 (3H, *m*), 7.09 (1H, dd, *J*** **=** **8.3, 1.8 Hz), 7.66 (3H, *s*) ppm. ^13^C NMR (100 MHz, CDCl_3_) *δ* = 204.1, 163.3 (d, *J*** **=** **248.5 Hz), 162.8, 148.2, 135.6 (d, *J*** **=** **3.3 Hz), 131.4, 129.1 (d, *J*** **=** **8.3 Hz), 118.7, 117.8, 116.4, 116.1 (d, *J*** **=** **21.6 Hz), 26.7 ppm. HRMS (ESI) [M + H]^+^: *m*/*z* calcd for (C_14_H_12_O_2_F) 231.0821. Found 231.0826. GC-MS (*m/z*, %): 215 (100), 230 (45).

#### 3-Hydroxy-4'-methoxy-1,1'-biphenyl-4-carbaldehyde (4h)^39^


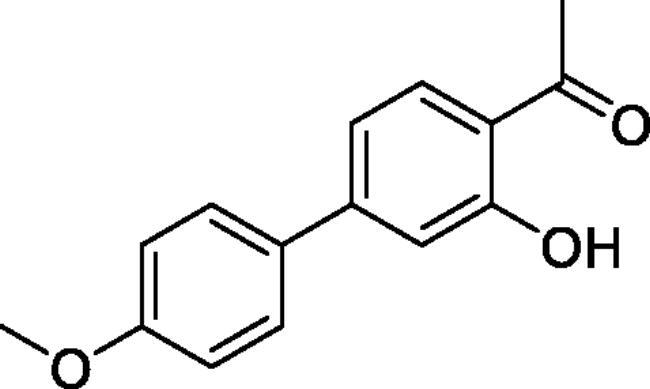


Obtained from 4′-bromo-2′-hydroxyacetophenone (0.50 g, 2.33 mmol), 4-methoxybenzeneboronic acid (0.46 g, 3.02 mmol), K_2_CO_3_ (0.80 g, 5.81 mmol) and Pd(PPh_3_)_4_ (0.13 g, 0.12 mmol). Obtained as white solid (0.31 g, 74%). Mp 124–125 °C. IR (KBr) cm^−1^ = 2969 (OH), 1635 (C = O). ^1^H NMR (400 MHz, CDCl_3_) *δ* = 12.37 (1H, *s*), 7.76 (1H, d, *J*** **=** **8.3 Hz), 7.58 (2H, d, *J*** **=** **8.9 Hz), 7.17 (1H, d, *J*** **=** **1.8 Hz), 7.12 (1H, dd, *J*** **=** **8.3, 1.8 Hz), 6.99 (2H, d, *J*** **=** **8.9 Hz), 3.86 (3H, *s*), 2.65 (3H, *s*) ppm. ^13^C NMR (100 MHz, CDCl_3_) *δ* = 204.0, 162.9, 160.4, 148.9, 131.8, 131.3, 128.5, 118.3, 117.5, 115.7, 114.5, 55.5, 26.7 ppm. HRMS (ESI) [M + H]^+^: *m*/*z* calcd for (C_15_H_15_O_3_) 243.1021. Found 243.1028. GC-MS (*m/z*, %): 227 (100), 242 (56).

#### 3',4'-Dichloro-3-hydroxy-1,1'-biphenyl-4-carbaldehyde (4i)


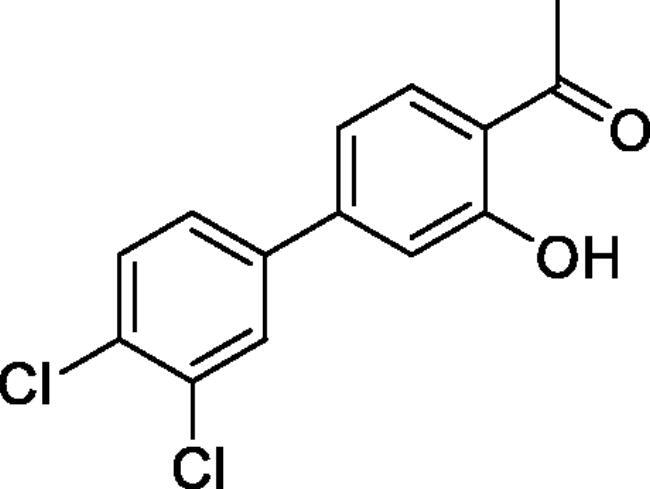


Obtained from 4′-bromo-2′-hydroxyacetophenone (0.50 g, 2.33 mmol), 3,4-dichlorobenzeneboronic acid (0.58 g, 3.02 mmol), K_2_CO_3_ (0.80 g, 5.81 mmol) and Pd(PPh_3_)_4_ (0.13 g, 0.12 mmol). Obtained as white solid (0.41 g, 61%). Mp 166–167 °C. IR (KBr) cm^−1^ = 1634 (C = O). ^1^H NMR (400 MHz, CDCl_3_) *δ* = 12.3 (1H, *s*), 7.81 (1H, d, *J*** **=** **8.3 Hz), 7.70 (1H, dd, *J*** **=** **8.3, 2.1 Hz), 7.53 (1H, d, *J*** **=** **8.3 Hz), 7.44 (1H, dd, *J*** **=** **8.3, 2.1 Hz), 7.16 (1H, d, *J*** **=** **1.9 Hz), 7.08 (1H, dd, *J*** **=** **8.3, 1.9 Hz), 2.67 (3H, *s*) ppm. ^13^C NMR (100 MHz, CDCl_3_) *δ* = 204.2, 162.8, 146.5, 139.5, 133.3, 133.1, 131.5, 131.0, 129.2, 126.5, 119.2, 117.7, 116.6, 26.8 ppm. HRMS (ESI) [M - H]^-^: *m*/*z* calcd for (C_14_H_9_O_2_Cl) 278.9980. Found 278.9980. GC-MS (*m/z*, %): 265 (100), 267 (65), 280 (37).

#### Ethyl 4'-formyl-3'-hydroxy-1,1'-biphenyl-4-carboxylate (4j)


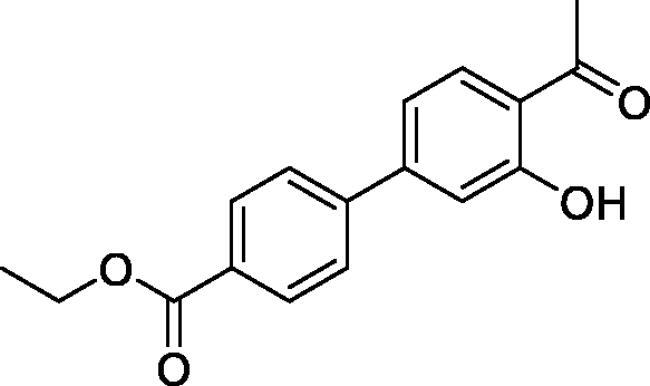


Obtained from 4′-bromo-2′-hydroxyacetophenone (0.50 g, 2.33 mmol), 4-(ethoxycarbonyl)benzeneboronic acid (0.65 g, 3.02 mmol), K_2_CO_3_ (0.80 g, 5.81 mmol) and Pd(PPh_3_)_4_ (0.13 g, 0.12 mmol). Obtained as white solid (0.41 g, 61%). Mp 106–107 °C. IR (KBr) cm^−1^ = 2996 (OH), 1704 (C = O), 1642 (C = O). ^1^H NMR (400 MHz, CDCl_3_) *δ* = 12.30 (1H, *s*), 8.13 (2H, d, *J*** **=** **8.4 Hz), 7.82 (1H, d, *J*** **=** **8.4 Hz), 7.68 (2H, d, *J*** **=** **8.4 Hz), 7.24 (1H, d, *J*** **=** **1.8 Hz), 7.17 (1H, dd, *J*** **=** **8.4, 1.8 Hz), 4.14 (2H, q, *J*** **=** **7.2 Hz), 2.67 (3H, *s*), 1.42 (3H, *t*, *J*** **=** **7.2 Hz) ppm. ^13^C NMR (100 MHz, CDCl_3_) *δ* = 204.2, 166.4, 162.8, 148.0, 143.7, 131.4, 130.6, 130.3, 127.3, 119.2, 118.1, 117.0, 61.3, 26.8, 14.5 ppm. HRMS (ESI) [M + H]^+^: *m*/*z* calcd for (C_17_H_17_O_4_) 285.1127. Found 285.1135. GC-MS (*m/z*, %): 241 (27), 269 (10), 284 (56).

#### General procedure for the preparation of 1,2,3-benzoxathiazine 2,2-dioxides 5a-j

The derivative of 1–(2-hydroxyphenyl)ethanone **4a-j** (1 equiv) was dissolved in dry DMA (6 ml). Reaction mixture was cooled to 0 °C. Sulphamoyl chloride (2.5 equiv) was slowly added to the reaction mixture. The stirring was continued at room temperature under argon atmosphere for 24 h. Reaction mixture was then poured into ice-water (25 ml), extracted with DCM (3 × 25 ml), washed with satd. NaHCO_3_ (3 × 25 ml) and satd. NaCl (3 × 25 ml). Organic phase was dried over Na_2_SO_4_, filtered, evaporated. The product was purified by column chromatography with PE/EtOAc (2:1) followed by recrystallization from EtOH.

#### 4-Methyl-6-phenyl-1,2,3-benzoxathiazine 2,2-dioxide (5a)


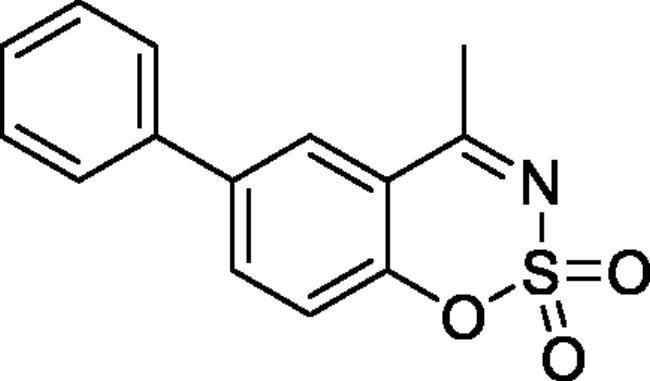


Obtained from 4-hydroxy-1,1′-biphenyl-3-carbaldehyde (**4a**)(0.20 g, 0.94 mmol) and sulphamoyl chloride(0.27 g, 2.36 mmol). Obtained as light brown (0.08 g, 29%). Mp 147–148 °C. IR (KBr) cm^−1^ = 1383 (S = O), 1197 (S = O), 1187 (S = O). ^1^H NMR (400 MHz, CDCl_3_) *δ* = 7.88–7.93 (2H, *m*), 7.42–7.58 (5H, *m*), 7.37 (1H, dd, *J*** **=** **8.4, 0.4 Hz), 2.78 (3H, *s*) ppm. ^13^C NMR (100 MHz, CDCl_3_) *δ* = 177.5, 152.8, 139.7, 138.6, 135.9, 129.4, 128.6, 127.3, 126.9, 119.5, 116.7, 23.9 ppm. HRMS (ESI) [M + H]^+^: *m*/*z* calcd for (C_14_H_12_NO_3_S) 274.0538. Found 274.0540. GC-MS (*m/z*, %): 139 (87), 140 (45), 209 (88), 273 (100).

#### 6–(4-Fluorophenyl)-4-methyl-1,2,3-benzoxathiazine 2,2-dioxide (5 b)


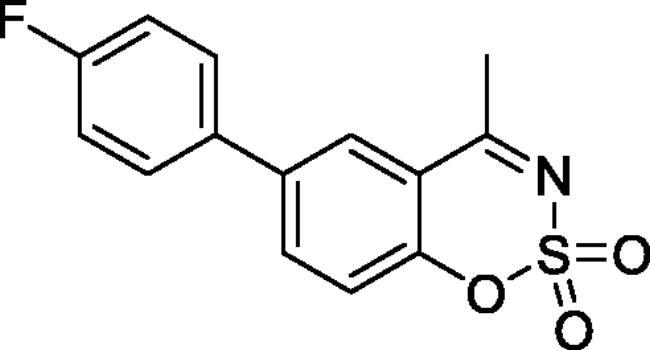


 Obtained from 4′-fluoro-4-hydroxy-1,1′-biphenyl-3-carbaldehyde (**4** **b**) (0.25 g, 1.09 mmol) and sulphamoyl chloride (0.31 g, 2.72 mmol). Obtained as brown solid (0.14 g, 45%). Mp 175–176 °C. IR (KBr) cm^−1^ = 1378 (S = O), 1185 (S = O). ^1^H NMR (400 MHz, CDCl_3_) *δ* = 7.83–7.88 (2H, *m*), 7.51 (2H, dd, *J*** **=** **8.9, 5.2 Hz), 7.37 (1H, dd, *J*** **=** **8.4, 0.5 Hz), 7.19 (2H, t, *J*** **=** **8.7 Hz), 2.78 (3H, *s*) ppm. ^13^C NMR (100 MHz, CDCl_3_) *δ* = 177.4, 163.2 (d, *J*** **=** **249.3 Hz), 152.8, 138.7, 135.8, 134.8 (d, *J*** **=** **3.2 Hz), 129.0 (d, *J*** **=** **8.2 Hz), 126.7, 119.7, 116.8, 116.4 (d, *J*** **=** **21.8 Hz), 23.9 ppm. HRMS (ESI) [M + H]^+^: *m*/*z* calcd for (C_14_H_11_NO_3_SF) 292.0444. Found 292.0445. GC-MS (*m/z*, %): 157 (80), 158 (49), 227 (91), 291 (100).

#### 6–(4-Methoxyphenyl)-4-methyl-1,2,3-benzoxathiazine 2,2-dioxide (5c)


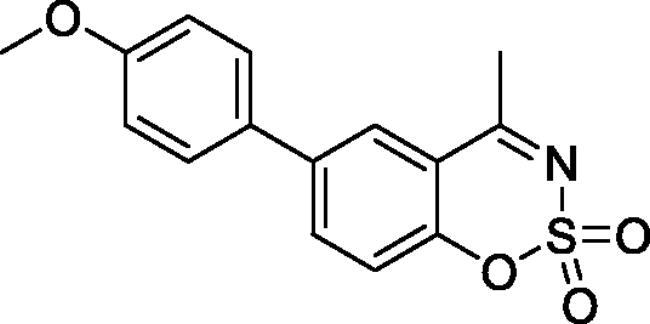


Obtained from 4-hydroxy-4′-methoxy-1,1′-biphenyl-3-carbaldehyde (**4c**) (0.25 g, 1.03 mmol) and sulphamoyl chloride(0.30 g, 2.58 mmol). Obtained as yellow solid (0.13 g, 41%). Mp 121–122 °C. IR (KBr) cm^−1^ = 1386 (S = O), 1375 (S = O), 1189 (S = O). ^1^H NMR (400 MHz, CDCl_3_) *δ* = 7.83 (2H, *m*), 7.48 (2H, d, *J*** **=** **8.9 Hz), 7.34 (1H, dd, *J*** **=** **8.2, 0.8 Hz), 7.02 (2H, d, *J*** **=** **8.9 Hz), 3.87 (3H, s), 2.77 (3H, *s*) ppm. ^13^C NMR (100 MHz, CDCl_3_) *δ* = 177.6, 160.2, 152.3, 139.4, 135.5, 131.0, 128.4, 126.3, 119.5, 116.7, 114.8, 55.6, 23.9 ppm. HRMS (ESI) [M + H]^+^: *m*/*z* calcd for (C_15_H_14_NO_4_S) 304.0644. Found 304.0654.

#### 6–(3,4-Dichlorophenyl)-4-methyl-1,2,3-benzoxathiazine 2,2-dioxide (5d)


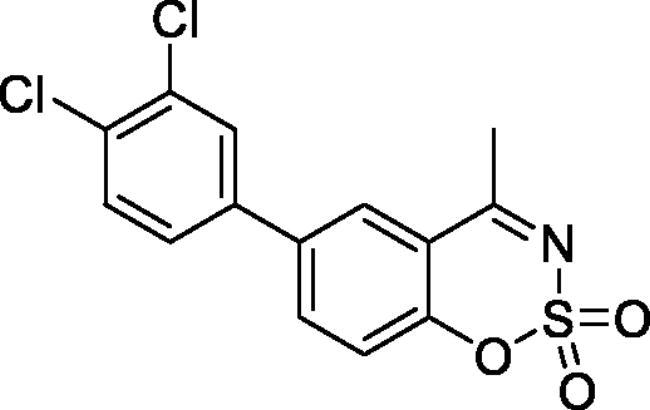


Obtained from 3′,4′-dichloro-4-hydroxy-1,1′-biphenyl-3-carbaldehyde (**4d**) (0.22 g, 0.78 mmol) and sulphamoyl chloride(0.23 g, 1.96 mmol). Obtained as white solid (0.06 g, 22%). Mp 130–131 °C. IR (KBr) cm^−1^ = 1388 (S = O), 1190 (S = O). ^1^H NMR (400 MHz, CDCl_3_) *δ* = 7.83–7.89 (2H, *m*), 7.63 (1H, d, *J*** **=** **2.2 Hz), 7.58 (1H, d, *J*** **=** **8.4 Hz), 7.39 (1H, dd, *J*** **=** **8.4, 0.5 Hz), 7.37 (1H, dd, *J*** **=** **8.4, 2.2 Hz), 2.80 (3H, *s*) ppm. ^13^C NMR (100 MHz, CDCl_3_) *δ* = 177.2, 153.3, 138.5, 137.2, 135.6, 133.7, 133.2, 131.4, 129.1, 126.8, 126.5, 120.0, 116.9, 24.0 ppm. HRMS (ESI) [M + H]^+^: *m*/*z* calcd for (C_14_H_8_NO_3_SCl) 339.9602. Found 339.9608.

#### Ethyl 4–(4-methyl-2,2-dioxido-1,2,3-benzoxathiazin-6-yl)benzoate (5e)


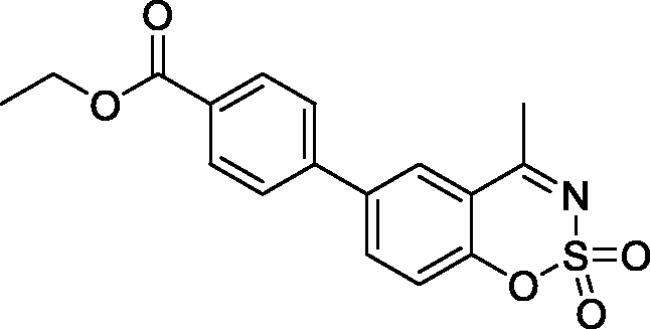


Obtained from ethyl 3′-formyl-4′-hydroxy-1,1′-biphenyl-4-carboxylate (**4e**) (0.22 g, 0.77 mmol) and sulphamoyl chloride(0.22 g, 1.94 mmol). Obtained as white solid (0.10 g, 37%). Mp 190–191 °C. IR (KBr) cm^−1^ = 1384 (S = O), 1369 (S = O), 1193 (S = O), 1182 (S = O). ^1^H NMR (400 MHz, CDCl_3_) *δ* = 8.17 (2H, d, *J*** **=** **8.6 Hz), 7.91–7.97 (2H, *m*), 7.62 (2H, d, *J*** **=** **8.6 Hz), 7.40 (1H, d, *J*** **=** **8.5 Hz), 4.42 (2H, q, *J*** **=** **7.2 Hz), 2.80 (3H, *s*), 1.43 (3H, *t*, *J*** **=** **7.2 Hz) ppm. ^13^C NMR (100 MHz, CDCl_3_) *δ* = 177.3, 166.2, 153.3, 142.7, 138.5, 135.9, 130.5, 130.6, 127.2, 127.1, 119.8, 116.8, 61.4, 24.0, 14.5 ppm. HRMS (ESI) [M + H]^+^: *m*/*z* calcd for (C_17_H_16_NO_5_S) 346.0749. Found 346.0747.

#### 4-Methyl-7-phenyl-1,2,3-benzoxathiazine 2,2-dioxide (5f)


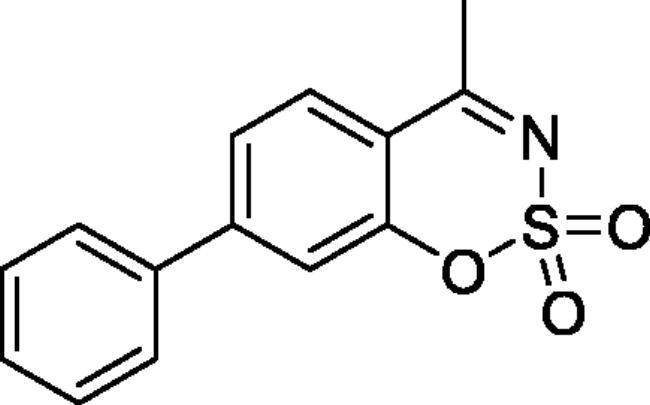


Obtained from 3-hydroxy-1,1′-biphenyl-4-carbaldehyde (**4f**) (0.20 g, 0.94 mmol) and sulphamoyl chloride (0.27 g, 2.36 mmol). Obtained as light yellow solid (0.11 g, 43%). Mp 181–182 °C. IR (KBr) cm^−1^ = 1378 (S = O), 1369 (S = O), 1188 (S = O), 1154 (S = O). ^1^H NMR (400 MHz, CDCl_3_) *δ* = 7.84 (1H, d, *J*** **=** **8.3 Hz), 7.58–7.65 (3H, *m*), 7.45–7.55 (4H, *m*), 2.75 (3H, *s*) ppm. ^13^C NMR (100 MHz, CDCl_3_) *δ* = 177.1, 154.1, 150.5, 137.9, 129.8, 129.5, 129.0, 127.4, 124.5, 117.2, 115.1, 23.8 ppm. HRMS (ESI) [M + H]^+^: *m*/*z* calcd for (C_14_H_12_NO_3_S) 274.0538. Found 274.0536. GC-MS (*m/z*, %): 139 (45), 180 (25), 209 (30), 273 (100).

#### 7–(4-Fluorophenyl)-4-methyl-1,2,3-benzoxathiazine 2,2-dioxide (5g)


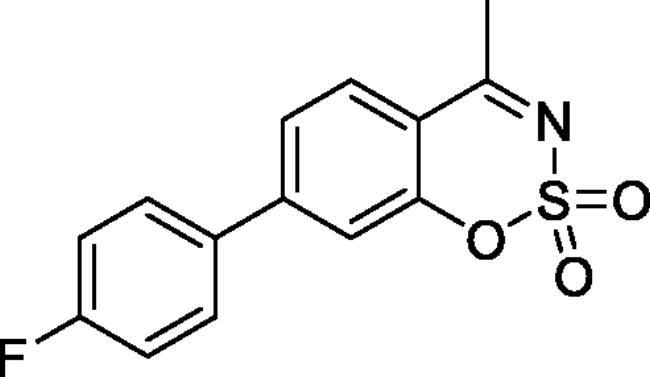


Obtained from 4′-fluoro-3-hydroxy-1,1′-biphenyl-4-carbaldehyde (**4** **g**) (0.22 g, 0.96 mmol) and sulphamoyl chloride (0.28 g, 2.39 mmol). Obtained as white solid (0.14 g, 49%). Mp 201–202 °C. IR (KBr) cm^−1^ = 1375 (S = O), 1189 (S = O), 1157 (S = O). ^1^H NMR (400 MHz, CDCl_3_) *δ* = 7.84 (1H, d, *J*** **=** **8.3 Hz), 7.61 (2H, dd, *J*** **=** **8.9, 5.2 Hz), 7.55 (1H, dd, *J*** **=** **8.3, 1.8 Hz), 7.44 (1H, d, *J*** **=** **1.8 Hz), 7.20 (2H, t, *J*** **=** **8.6 Hz), 2.75 (3H, *s*) ppm. ^13^C NMR (100 MHz, CDCl_3_) *δ* = 177.1, 163.9 (d, *J*** **=** **250.3 Hz), 154.1, 149.3, 134.1 (d, *J*** **=** **3.4 Hz), 129.3 (d, *J*** **=** **8.5 Hz), 129.1, 124.3, 117.0, 116.6 (d, *J*** **=** **21.8 Hz), 115.1, 23.8 ppm. HRMS (ESI) [M + H]^+^: *m*/*z* calcd for (C_14_H_11_NO_3_SF) 292.0444. Found 292.0457. GC-MS (*m/z*, %): 157 (41), 198 (25), 227 (28), 291 (100).

#### 7–(4-Methoxyphenyl)-4-methyl-1,2,3-benzoxathiazine 2,2-dioxide (5h)


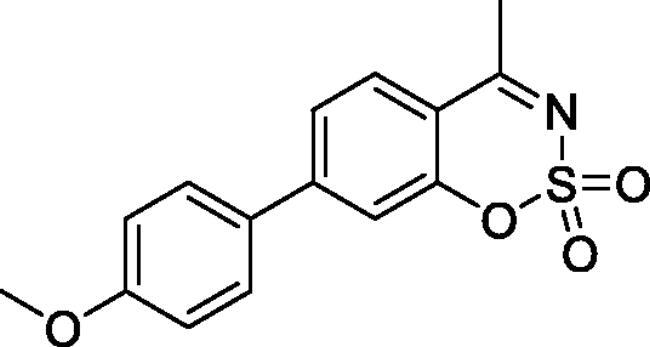


Obtained from 3-hydroxy-4′-methoxy-1,1′-biphenyl-4-carbaldehyde (**4** **h**) (0.22 g, 0.91 mmol) and sulphamoyl chloride (0.26 g, 2.27 mmol). Obtained as light brown solid (0.11 g, 38%). Mp 182–183 °C. IR (KBr) cm^−1^ = 1378 (S = O), 1363 (S = O), 1197 (S = O). ^1^H NMR (400 MHz, CDCl_3_) *δ* = 7.80 (1H, d, *J*** **=** **8.3 Hz), 7.59 (2H, d, *J*** **=** **8.9 Hz), 7.56 (1H, dd, *J*** **=** **8.3, 1.8 Hz), 7.44 (1H, d, *J*** **=** **1.8 Hz), 7.03 (2H, d, *J*** **=** **8.8 Hz), 3.88 (3H, *s*), 2.73 (3H, *s*) ppm. ^13^C NMR (100 MHz, CDCl_3_) *δ* = 177.1, 161.2, 154.2, 150.1, 130.1, 129.0, 128.7, 123.8, 116.2, 114.9, 114.5, 55.6, 23.8 ppm. HRMS (ESI) [M + H]^+^: *m*/*z* calcd for (C_15_H_14_NO_4_S) 304.0644. Found 304.0656.

#### 7–(3,4-Dichlorophenyl)-4-methyl-1,2,3-benzoxathiazine 2,2-dioxide (5i)


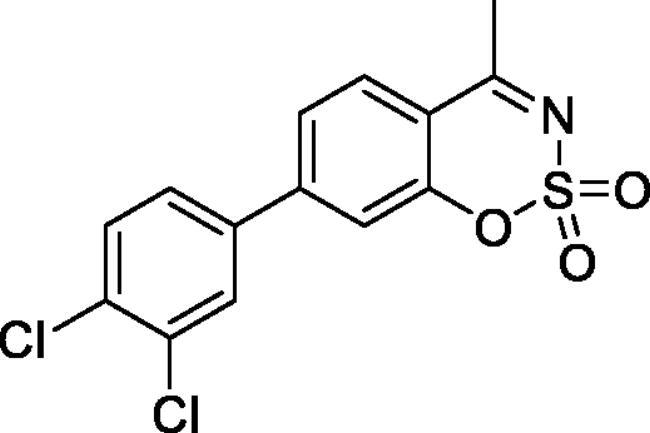


Obtained from 3′,4′-dichloro-3-hydroxy-1,1′-biphenyl-4-carbaldehyde (**4i**) (0.22 g, 0.78 mmol) and sulphamoyl chloride(0.23 g, 1.96 mmol). Obtained as white solid (0.11 g, 42%). Mp 202–203 °C. IR (KBr) cm^−1^ = 1370 (S = O), 1196 (S = O), 1178 (S = O). ^1^H NMR (400 MHz, CDCl_3_) *δ* = 7.86 (1H, d, *J*** **=** **8.4 Hz), 7.72 (1H, d, *J*** **=** **2.2 Hz), 7.59 (1H, d, *J*** **=** **8.4 Hz), 7.55 (1H, dd, *J*** **=** **8.3, 1.8 Hz), 7.44–7.48 (2H, *m*), 2.76 (3H, *s*) ppm. ^13^C NMR (100 MHz, CDCl_3_) *δ* = 176.9, 154.1, 147.7, 137.8, 134.3, 133.9, 131.5, 129.3, 129.2, 126.6, 124.3, 117.2, 115.8, 23.9 ppm. HRMS (ESI) [M - H]^-^: *m*/*z* calcd for (C_14_H_8_NO_3_SCl_2_) 339.9602. Found 339.9600.

#### Ethyl 4–(4-methyl-2,2-dioxido-1,2,3-benzoxathiazin-7-yl)benzoate (5j)


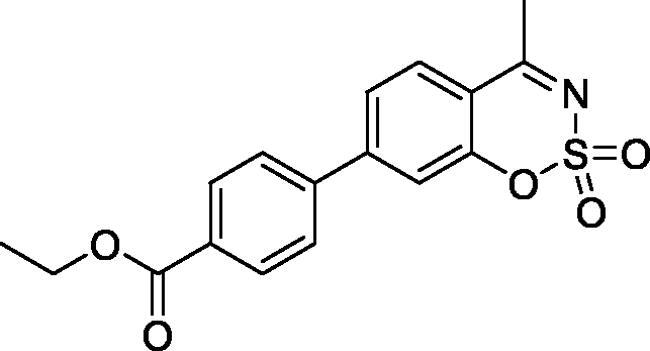


Obtained from ethyl 4′-formyl-3′-hydroxy-1,1′-biphenyl-4-carboxylate (**4j**) (0.22 g, 0.77 mmol) and sulphamoyl chloride (0.22 g, 1.94 mmol). Obtained as white solid (0.10 g, 38%). Mp 151–152 °C. IR (KBr) cm^−1^ = 1387 (S = O), 1198 (S = O). ^1^H NMR (400 MHz, CDCl_3_) *δ* = 8.18 (2H, d, *J*** **=** **8.7 Hz), 7.88 (1H, d, *J*** **=** **8.3 Hz), 7.69 (2H, d, *J*** **=** **8.7 Hz), 7.63 (1H, dd, *J*** **=** **8.3, 1.7 Hz), 7.52 (1H, d, *J*** **=** **1.7 Hz), 4.42 (2H, q, *J*** **=** **7.2 Hz), 2.76 (3H, *s*), 1.43 (3H, *t*, *J*** **=** **7.2 Hz) ppm. ^13^C NMR (100 MHz, CDCl_3_) *δ* = 177.0, 166.0, 154.1, 149.2, 142.0, 131.6, 130.6, 129.1, 127.4, 124.7, 117.5, 115.7, 61.5, 23.9, 14.5 ppm. HRMS (ESI) [M + H]^+^: *m*/*z* calcd for (C_17_H_16_NO_5_S) 346.0749. Found 346.0759.

### Ca inhibitory assay

An applied photophysics stopped-flow instrument has been used for assaying the CA catalysed CO_2_ hydration activity^40^.

Phenol red (at a concentration of 0.2 mM) was used as indicator, working at the absorbance maximum of 557 nm, with 20 mM Hepes (pH 7.5), and 20 mM Na_2_SO_4_ (for maintaining constant the ionic strength), following the initial rates of the CA-catalysed CO_2_ hydration reaction for a period of 10 – 100 s. The CO_2_ concentrations ranged from 1.7 to 17 mM for the determination of the kinetic parameters and inhibition constants. For each inhibitor, at least six traces of the initial 5 – 10% of the reaction have been used for determining the initial rate. The uncatalysed rates were determined in the same manner and subtracted from the total observed rates. Stock solutions of inhibitor (0.1 mM) were prepared in distilled – deionised water, and dilutions up to 0.01 nM were done thereafter with the assay buffer. Inhibitor and enzyme solutions were preincubated together for 15 min at room temperature prior to assay in order to allow for the formation of the E – I complex. Data from Table 1 were obtained after 6 h incubation of enzyme and inhibitor. The inhibition constants were obtained by nonlinear least-squares methods using PRISM 3 and the Cheng – Prusoff equation, as reported earlier^41–46^ and represent the mean from at least three different determinations. All CA isoforms were recombinant ones obtained in-house as reported earlier^15^^,^^47–55^.

**Table 1.** Inhibition data of human CA isoforms hCA I, II, IX, XII with compounds **2a-j** and **5a-j** reported here and the standard inhibitor acetazolamide (AAZ) by a stopped flow CO_2_ hydrase assay.

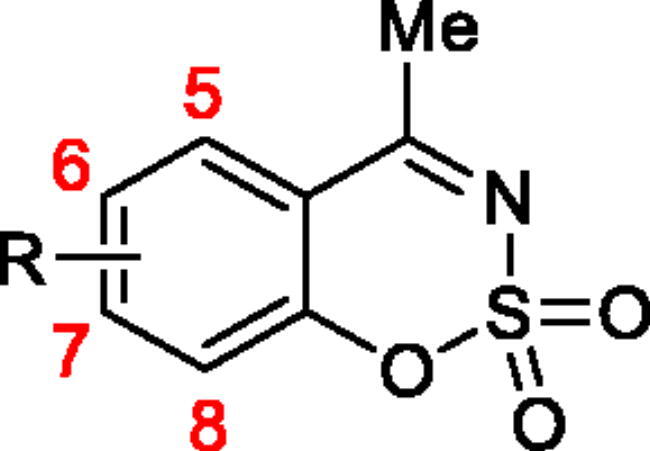					
cmp	R	K_I_ (nM)^a,b^			
		hCA I	hCA II	hCA IX	hCA XII
**2a**	5-Ome	>100000	>100000	75320	>100000
**2b**	6-Me	21020	456.8	72.1	42.0
**2c**	6-F	12360	228.7	53.4	15.7
**2d**	6-Cl	45690	634.1	49.1	29.6
**2e**	6-Br	>100000	5568	25.8	116.2
**2f**	7-Me	33210	788.2	48.0	34.8
**2g**	7-Ome	24890	558.6	63.4	22.9
**2h**	7-F	10450	284.0	59.7	19.3
**2i**	7-Cl	82050	1659	75.9	78.1
**2j**	7-Br	>100000	11450	99.8	152.9
**5a**	6-Ph	60590	936.4	28.5	390.4
**5b**	6-(4-F-C_6_H_4_)	24510	1529	22.6	53.7
**5c**	6-(4-Ome-C_6_H_4_)	70513	7118	30.4	10.5
**5d**	6-(3,4-Cl_2_-C_6_H_3_)	>100000	4527	30.8	130.7
**5e**	6-(4-CO_2_Et-C_6_H_4_)	19630	8254	93.6	12.2
**5f**	7-Ph	>100000	2364	7.0	230.6
**5g**	7-(4-F-C_6_H_4_)	>100000	5374	52.7	13.8
**5h**	7-(4-Ome-C_6_H_4_)	>100000	3165	57.3	180.4
**5i**	7-(3,4-Cl_2_-C_6_H_3_)	>100000	10020	42.1	15.6
**5j**	7-(4-CO_2_Et-C_6_H_4_)	>100000	6781	12.9	52.0
**AAZ**	–	250.0	12.5	25.0	5.7

^a^Mean from 3 different assays, by a stopped flow technique (errors were in the range of ± 5–10% of the reported values); b. 15 min incubation.

## Results and discussion

### Chemistry

**Scheme 1.**
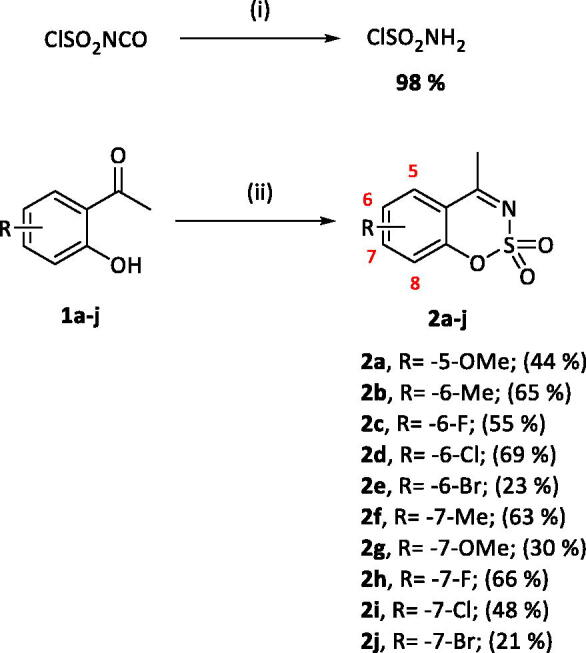
Reagents and conditions: (i). HCOOH, 0 °C, 15 min, then RT, 45 min. (ii). ClSO_2_NH_2_, DMA, RT, 24 h.

A series of 4-methylbenzo[1,2,3]oxathiazine-2,2-dioxides **2a-j** was prepared from corresponding aryl 2-hydroxybenzaldehydes **1a-j** in their reaction with sulphamoyl chloride^47^^,^^56^ that was prepared from chlorosulphonyl isocyanate (Scheme 1). The yields of the products were moderate (Table 1).

**Scheme 2.**
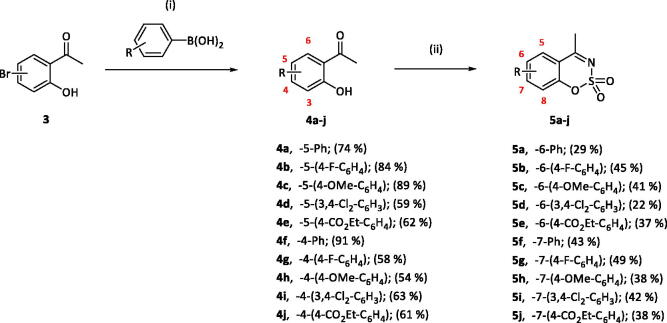
Reagents and conditions: (i). K_2_CO_3_, Pd(PPh_3_)_4_, toluene/H_2_O (5:1), 90 °C, 24 h. (ii). ClSO_2_NH_2_, DMA, RT, 24 h.

5-, 7- and 8-aryl substituted 4-methylbenzo[1,2,3]oxathiazine-2,2-dioxides **5a-j** were obtained from aldehydes **4a-j** (Scheme 2). The first step was Suzuki-Miyaura coupling of compounds **3** with various boronic acids followed by cyclisation using sulfamoylphchloride. Yields of the intermediates were from moderate to high but yields of the products are quite low due to the loss during purification process.

### Carbonic anhydrase inhibition

20 derivatives of 4-methyl-1,2,3-benzoxathiazine 2,2-dioxide were screened for the inhibition of four human CA isoforms – the cytosolic off-target hCA I and II as well as transmembrane, tumour-associated hCA IX and XII that are anticancer drug targets^15^^,^^57–62^. Inhibition data of 4-methyl-1,2,3-benzoxathiazine 2,2-dioxides **2a-j** and **5a-j** (as well as acetazolamide AAZ as standard) against hCA I, II, IX and XII, after 6 h of incubation period of the enzyme and inhibitor solutions have been reported (Table 1)^40^.

None of screened derivatives showed significant inhibitory activity towards off-target hCA I. 7-Aryl substituted compounds **5f-j** as well as some others (**2a**, **2e**, **2j**, **5d**) do not inhibit hCA I at all, whereas other ones showed micromolar inhibitory activity (*K*_I_ = 10 – 82 µM).

Compound **2a** was the only one which did not inhibit cytosolic off-target hCA II. Other ones showed slight inhibitory properties. In general, aryl substituted derivatives inhibited hCA II less effectively than compounds **2a-j** with small substituents in positions 5, 6 or 7.

Target transmembrane isoenzyme hCA IX was significantly inhibited by most derivatives in nanomolar range (*K*_I_ = 7.0 − 99.8 nM), except compound **2a** which *K*_I_ = 75 µM.

Another target tumour-associated isoenzyme hCA XII was effectively inhibited by most derivatives (*K*_I_ = 10.5 – 390 nM) as well, although compound **2a** did not express hCA XII inhibitory properties also in this case.

Compound **2a** was the only one which showed very low or did not show any inhibitory activity against target as well as non-target hCAs. This is probably due to the sterical hindrance provided by the substituent in position 5. Perhaps 5-methoxy group limits binding of the compound **2a** to the enzyme.

In general 4-methyl-1,2,3-benzoxathiazine 2,2-dioxides exhibited excellent selectivity towards hCA IX/XII over hCA I and very good selectivity towards hCA IX/XII over hCA II except compound **2a**. Some of the most promising derivatives that express selectivity towards both off-target hCA isoforms are aryl substituted 4-methyl-1,2,3-benzoxathiazine 2,2-dioxides **5c**, **5e**, **5i**, as well as Br- substituted ones (**2e**, **2j**).

## Conclusions

Here we report a series of 4-methyl-1,2,3-benzoxathiazine-2,2-dioxide derivatives **2a-j** with small substituents (Me, MeO, F, Br) in 5, 6 or 7 position prepared by straightforward synthesis from corresponding 2-hydroxyacetophenones in their reaction with sulphamoyl chloride. A series of 6- or 7-aryl substituted 4-methyl-1,2,3-benzoxathiazine-2,2-dioxides **5a-j** was obtained in two-step protocol from aryl substituted 2-hydroxyacetophenones in Suzuki-Miyaura reaction with various boronic acids followed by cyclisation using sulphamoyl chloride.

The derivatives were assayed as inhibitors of four hCA isoforms, the cytosolic hCA I and II, and the transmembrane, tumour-associated hCA IX and XII.

Only compound **2a** did not inhibit any of investigated hCA isoforms.

4-Methyl-1,2,3-benzoxathiazine-2,2-dioxides generally do not inhibit or show low inhibitory activity towards cytosolic off-target hCA I.

Off-target hCA II was slightly inhibited by the derivatives. Better inhibition of hCA II was observed in the case of 4-methyl-1,2,3-benzoxathiazine-2,2-dioxides without aryl substituents.

Transmembrane target isoform hCA IX was significantly inhibited by most derivatives in nanomolar range.

Another tumour-associated target isoform hCA XII was effectively inhibited by almost all derivatives as well.

Excellent selectivity towards hCA IX/XII over hCA I and very good selectivity towards hCA IX/XII over hCA II was observed in most cases of 4-methyl-1,2,3-benzoxathiazine-2,2-dioxide derivatives.
